# MiR-675 Inhibits Primary Ovarian Tumor Growth and Metastasis by Suppressing EMT and TGFβ Signaling

**DOI:** 10.7150/jca.102654

**Published:** 2025-01-01

**Authors:** Xinxin Zhao, Ziping Liu, Yongshuai Li, Shiji Song, Baojin Wang, Lawrence M. Pfeffer, Wenjing Zhang, Junming Yue

**Affiliations:** 1 Department of Gynecology and Obstetrics, the Third Affiliated Hospital of Zhengzhou University, Zhengzhou, Henan, 450052, China.; 2 Department of Pathology and Laboratory Medicine, College of Medicine, the University of Tennessee Health Science Center, Memphis, TN, 38163, USA.; 3 Center for Cancer Research, College of Medicine, the University of Tennessee Health Science Center, Memphis, TN, 38163, USA.; 4 Department of Thyroid Surgery, the First Affiliated Hospital of Zhengzhou University, Zhengzhou, Henan, 450052, China.; 5 Department of Genetics, Genomics & Informatics, Collage of Medicine, University of Tennessee Health Science Center, Memphis, TN, 38163, USA.

**Keywords:** miR-675, ovarian cancer, EMT, TGFβ, orthotopic mouse model

## Abstract

MicroRNAs (miRNAs) can function as either tumor suppressors or oncogenes. This study explores the role of miR-675 in ovarian cancer (OC) using *in vitro* OC cell lines and an *in vivo* orthotopic mouse model. We demonstrate that miR-675 expression inhibits primary tumor growth and metastasis by targeting TGFβ1, suppressing epithelial to mesenchymal transition (EMT), and attenuating the TGFβ signaling pathway. Functional assays revealed significant inhibition of cell proliferation, migration, and invasion by miR-675. In addition, miR-675 synergistically enhanced the apoptotic effect of paclitaxel and carboplatin, suggesting potential for combination therapy of miRNA-675 with chemotherapeutic agents. *In vivo* studies using orthotopic injection of miR-675 expressing and control OC cells in NSG mice demonstrated significant inhibition of primary OC growth and metastasis. These findings indicate that miR-675 is a promising therapeutic target for OC treatment.

## Introduction

Ovarian cancer (OC) remains a formidable challenge due to its aggressive nature and high metastatic potential. Despite advancements in treatment, the overall 5-year survival rate for OC patients remains below 50% 1, 2. Identifying novel biomarkers and exploring new therapeutic approaches are crucial to improve patient outcome 3, 4. MicroRNAs (miRNAs) are critical regulators of gene expression, influencing fundamental processes like cell proliferation, apoptosis, migration, and invasion 5. Interestingly, miRNAs can function as either tumor suppressors or oncogenes depending on the specific cancer type 6. This unique regulatory capacity makes them promising targets for novel therapeutic strategies 7, 8. miRNA-based drugs are already undergoing clinical trials (Phase I and II) for various human diseases, including different cancers 9, 10. Targeting miRNAs using mimics or inhibitors offers a promising approach for cancer therapy 11, 12.

miR-675, located within the exon 1 of the long non-coding RNA (lncRNA) H19 on the reverse strand of the genome, exhibits context-dependent functions 13-16. Both miR-675 and lncRNA H19 can act as oncogenes or tumor suppressors depending on the specific cancer type 17. miR-675 function as an oncogene by targeting tumor suppressors Rb, CDC25A, DMTF1 and RUNX1 in various cancers including human colorectal cancer 17, 18, gastric cancer 19, 20, hepatocarcinoma 21, 22, glioma 23, breast cancer 24 and cutaneous squamous cell carcinoma (cSCC) 25, 26. However, miR-675 has also been shown to play a role as a tumor suppressor in prostate cancer and thyroid cancer by targeting TGFβ induced protein (TGFβI) 27 or MAPK 28, respectively.

While miR-675-3p has been linked to chemoresistance in OC 29, its function in OC metastasis remains poorly understood. Epithelial-to-mesenchymal transition (EMT) contributes to tumor metastasis and chemoresistance in various cancers 30-32. However, the association of miR-675 expression with EMT is unclear across diverse cancer types. While some studies suggest its role in promoting EMT in pancreatic and colon cancers 33, 34 , its impact on EMT in OC remains unexplored.

Given the potential effect of miR-675 on EMT and tumor metastasis, this study aims to investigate its role in OC tumor progression and metastasis. We will utilize *in vitro* OC cell lines and an orthotropic *in vivo* OC mouse model to determine how miR-675 expression regulates EMT and contributes to primary tumor growth and metastasis. Additionally, we will evaluate the synergistic effect of miR-675 with paclitaxel and carboplatin, two chemotherapeutic agents, to assess its therapeutic potential for OC treatment.

## Materials and Methods

### Cell culture

OC cell lines OVCAR3 and OVCAR8 were purchased from National Cancer Institute and cultured as described before 35. Briefly, RPMI 1640 medium supplemented with 10% FBS (Hyclone, Logan, UT), 100 U/ml penicillin, and 100μg/ml streptomycin (Invitrogen, Carlsbad, CA) was used. HEK293FT cells were purchased from Invitrogen and cultured in DMEM medium with 10% FBS, 100 U/ml penicillin, 100 µg/ml streptomycin, 1% glutamine, 1% nonessential amino acid, and 1 µg/ml geneticin.

### Lentiviral vector production and transduction

Lentiviral vector pEF1a-miR-675-EGFP vector was purchased from Applied Biological Materials Inc. (Richmond, Canada) and plenti-SMAD2/3/4-luc reporter vector was purchased from Biosciences. Lentiviral vectors were packaged in HEK293FT cells and purified through ultracentrifugation as previously reported 36. Both OVCAR3 and OVCAR8 cells were transduced with 10 MOI of plenti-EF1a-miR-675 or empty control vector and then selected with 5 µg/ml puromycin, and stable cell lines were established.

### Extraction of total RNA and detection of miRNA expression using real‑time RT‑PCR

Total RNA was extracted using TRIzol® RNA Isolation Reagents (Cat. No.15596026, Ambion, USA) following the manufacturer's protocol and miR-675 expression in both OC cell lines was detected using the SYBR Green-based real-time (RT)-PCR as described previously 36 using forward primers for miR-675-5p (5′-TGGTGCGGAGAGGGCCCACAGTG-3') and miR-675-3p (5′-CTGTATGCCCTCACCGCTCA-3').

### Cell proliferation assay

MTT assay: miR-675 and control OVCAR3 or OVCAR8 cells were plated into 96-well plates (3000/well) and cultured for different time points (day 1 to 4). 10 µl of MTT reagent (10 mg/ml) was added to each well and incubated for an additional 4 h. Subsequently, 200 μl DMSO was added to stop the reaction and the culture plates were incubated for additional 10 mins. The absorbance was measured at 570 nm wavelength.

Live cell imaging assay: OVCAR3 or OVCAR8 cells transduced with miR-675 and control lentiviral vectors were seeded into 96-well plates at a density of 3,000 cells per well. The plates were then incubated in the IncuCyte S3 live cell imager (Sartorius, Göttingen, Germany) for 4 days. Cell confluence was monitored and quantified using the IncuCyte software.

### Cell colony formation assay

miR-675 and control OVCAR3 and OVCAR8 cells (300 cells /well) were seeded into 6-well plates and the media were refreshed every 3-4 days. Cell colonies were fixed with methanol and stained with crystal violet and then counted.

### Cell migration and invasion assay

Cell migration and invasion assays were performed as previously described 37. Briefly, OVCAR3 or OVCAR8 cells transduced with miR-675 and control cells (3,000 cells/well) were seeded in the upper chamber of modified Transwell inserts (BD Falcon™, San Jose, CA) containing 300 µL of serum-free RPMI 1640 medium. The lower chamber was filled with RPMI 1640 medium supplemented with 10% FBS, acting as a chemoattractant. Cells that traversed the membrane to the lower chamber were fixed with methanol and stained with crystal violet. For invasion assays, cells that penetrated the Matrigel matrix in the upper chamber of Transwell chambers and reached the lower surface were fixed and stained with hematoxylin and eosin (H&E). Cells were imaged at 20x magnification from at least three randomly chosen fields using a light microscope. The images were then analyzed with ImageJ software for cell quantification and statistical analysis.

### Western blot

Western blot analysis was performed as previously described 38. Briefly, cell or tissue lysates (100 μg) were separated by SDS-PAGE and transferred to nitrocellulose membranes. The membranes were probed with primary antibodies against E-cadherin (1:1000, #3195S), N-cadherin (1:1000, #13116S), vimentin (1:1000, #5741S), Snail2 (1:1000, #9585S), cleaved-PARP (1:1000, #5625S), cleaved-caspase-3 (1:500, #9661S), total SMAD2/3 (1:1000, #8685S), cytokeratin-7 (1:4000, #ab181598), TGFβR2 (1:1000, #SC-17799), and GAPDH (1:1000, #sc-47724) (Cell Signaling Technology, Danvers, MA, USA) or pSMAD2 (1:1000, #AB3849-I, EMD Millipore Corporation, Merck KGaA, Darmstadt, Germany). After incubation with appropriate secondary antibodies conjugated to horseradish peroxidase (HRP), protein bands were visualized using enhanced chemiluminescence (ECL) detection reagents.

### Immunofluorescent staining

miR-675 and control OVCAR8 cells (1 x 10^4^ cells/well) were seeded onto 24-well plates and then treated with either vehicle or TGFβ (10 ng/mL) for an additional 24 hours. Cells were then fixed with cold methanol for 15 minutes, followed by permeabilization with 0.2% Triton X-100 in PBS for 10 minutes. To block non-specific antibody binding, they were incubated in 5% bovine serum albumin (BSA) in PBS for 1 hour. Subsequently, the cells were incubated overnight with primary antibodies against cytokeratin-7 and vimentin (1:200 dilution; Cell Signaling, Danvers, MA). Following washes with PBS containing Tween-20 (PBST), the cells were incubated for 1 hour at room temperature with a secondary antibody: goat anti-rabbit conjugated with Alexa Fluor 594 (1:500 dilution; Invitrogen, Carlsbad, CA). Cell nuclei were counterstained with DAPI (Vector Laboratories, Inc.; Burlingame, CA). Finally, images were captured using a fluorescence microscope (Nikon, San Diego, CA).

### SMAD-dependent reporter gene luciferase assay

To assess the effect of miR-675 on SMAD signaling, miR-675-expressing and control OVCAR3 or OVCAR8 cells were transduced with a lentiviral vector, pGF-SMAD2/3/4-mCMV-luciferase-EF1a-puro (System Biosciences, CA). This vector contains firefly luciferase reporter gene under the control of SMAD2/3/4 transcriptional response elements (TREs). Following transduction, cells were treated with either PBS or 10 ng/mL TGFβ for 24 hours. Luciferase activity was then measured using the Dual-Luciferase Reporter Assay System (Promega, Madison, WI, USA) to quantify SMAD signaling activation. The luciferase activity was normalized to control for variations in cell number.

### Orthotopic ovarian cancer mouse model

To investigate the effects of miR-675 expression on ovarian tumor growth and metastasis *in vivo*, miR-675 and control OVCAR8 cells (1 x 10^6^ cells/mouse) stably labeled with luciferase (OVCAR8-Luc2) were injected intrabursally into four-week-old immunocompromised NOD-SCID gamma (NSG) female mice. Tumor progression and metastasis were monitored weekly using the Xenogen bioluminescence imaging system as described before 39, 40. At four weeks post-injection, mice were euthanized. The primary ovarian tumors and any metastatic lesions were collected for further analysis through histological examination and western blot analysis. All animal procedures were conducted following the protocol (#23-0421) approved by the Institutional Animal Care and Use Committee (IACUC) of the University of Tennessee Health Science Center.

### Statistical analysis

Statistical analyses were performed on data obtained from at least three independent experiments, with each experiment run in triplicate. Results are presented as mean values ± standard error of mean (SEM) to represent variability within each group. To evaluate statistically significant differences between groups, Student's t-test was used for comparisons between two groups, while two-way ANOVA was employed for comparisons involving multiple groups 41, 42. A p-value threshold of less than 0.05 (p < 0.05) was considered statistically significant 43.

## Results

### miR-675-5p and miR-675-3p are differentially expressed in OC cells

**Figure 1:**
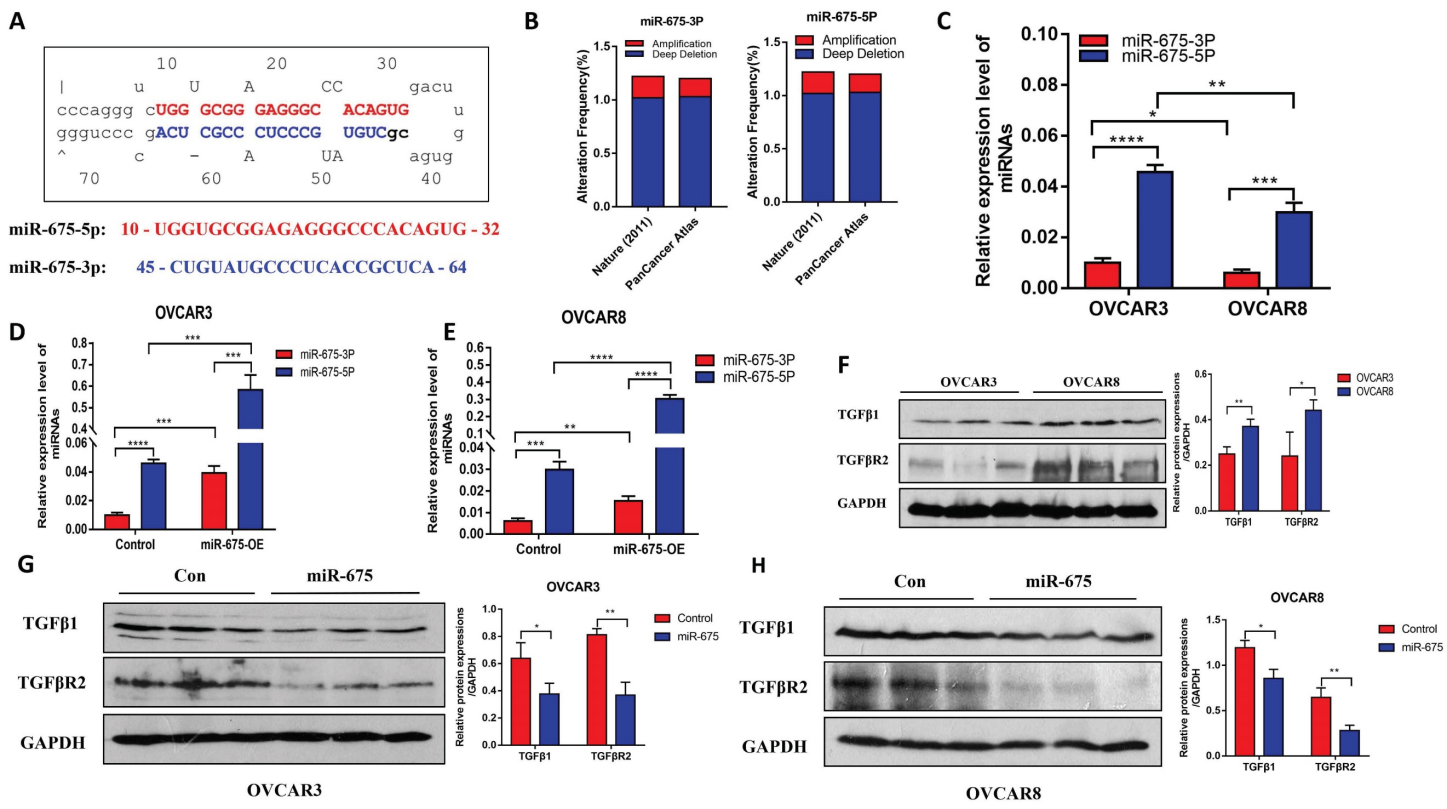
** miR-675 expression in OC cells and its effects on expression of TGFβ1 and TGFBR2. A,** miR-675 precursor hairpin structure and sequences of miR-675-5p and miR-675-3p**. B,** Gene amplification and deletions of miR-675-5p and miR-675-3p in OC patients from TCGA dataset. **C,** Endogenous expression of miR-675-5p and miR-675-3p in OC cells. **D&E,** miR-675-5p, and miR-675-3p expression in control and miR-675 transduced OVCAR3 cells (D) and OVCAR8 cells (E). **F,** Westen blot analysis of miR-675 targets TGFβ1 and downstream receptors in OC cells. **G&H,** miR-675 target TGFβ1 and TGFβR2 expression in OVCAR3 cells (G) and OVCAR8 cells (H). * *p* < 0.05; ** *p* < 0.01; *** *p* < 0.001; **** *p* < 0.0001.

miRNAs are transcribed in the cell nucleus as primary transcripts (pri-miRNAs), which are then processed into precursor miRNAs (pre-miRNAs) 44. These precursors are further matured in the cytoplasm by an enzyme complex called the RNA-induced silencing complex (RISC) to generate functional mature miRNAs 44. The miR-675 precursor gives rise to two mature miRNAs: miR-675-5p and miR-675-3p, derived from their origin from the 5' or 3' arm of the hairpin structure (Fig. 1A). To investigate the genetic alterations of miR-675 in OC, we analyzed data from The Cancer Genome Atlas (TCGA) database (https://www.cancer.gov/tcga). This analysis included 489 OC patients from a 2011 Nature dataset and 584 patients from the PanCancer Atlas dataset (Fig. 1B). Our analysis revealed alterations in either miR-675-5p or miR-675-3p in approximately 1.2% of patients from both datasets. Specifically, deletions of either miR-675-5p or miR-675-3p were found in ~1.0% of patients from these cohorts, respectively, while amplifications were identified in ~0.2% of patients (Fig. 1B). However, no point mutations were detected in the miR-675 sequences of any patients within these datasets. Next, we examined miR-675 expression levels in OC cell line and found that both miR-675-5p and miR-675-3p were more highly expression in the less invasive OVCAR3 cells as compared to the more invasive OVCAR8 cells (Fig. 1C). miR-675-3p expression was lower than miR-675-5p in both cell lines.

To investigate the functional role of miR-675, we established stable cell lines that over-express miR-675 by transducing OVCAR3 and OVCAR8 cells with lentiviral vectors expressing miR-675 or a control vector. We verified miR-675-5p and miR-675-3p expression in the transduced cells. Compared to control vector-transduced cells, miR-675-5p and miR-675-3p in OVCAR3 cells was over-expressed by approximately 12-fold and 4-fold, respectively (Fig. 1D), and in OVCAR8 cells by approximately 10-fold and 2-fold, respectively (Fig. 1E).

Previous studies suggest that miR-675 targets TGFβ1 and TGFBR1 by binding their 3'UTR 45-47. Thus, we examined the protein expression of TGFβ1, a target of miR-675 and its receptor TGFBR2, in both cell lines using western blotting. We observed differential expression of TGFβ1 and TGFBR2 protein between OVCAR3 and OVCAR8 cells, with higher expression in OVCAR8 cells (Fig. 1F). These two proteins were inhibited in both cell lines that over-express miR-675 (Fig. 1G and 1H).

### miR-675 expression inhibits EMT in OC cells

**Figure 2:**
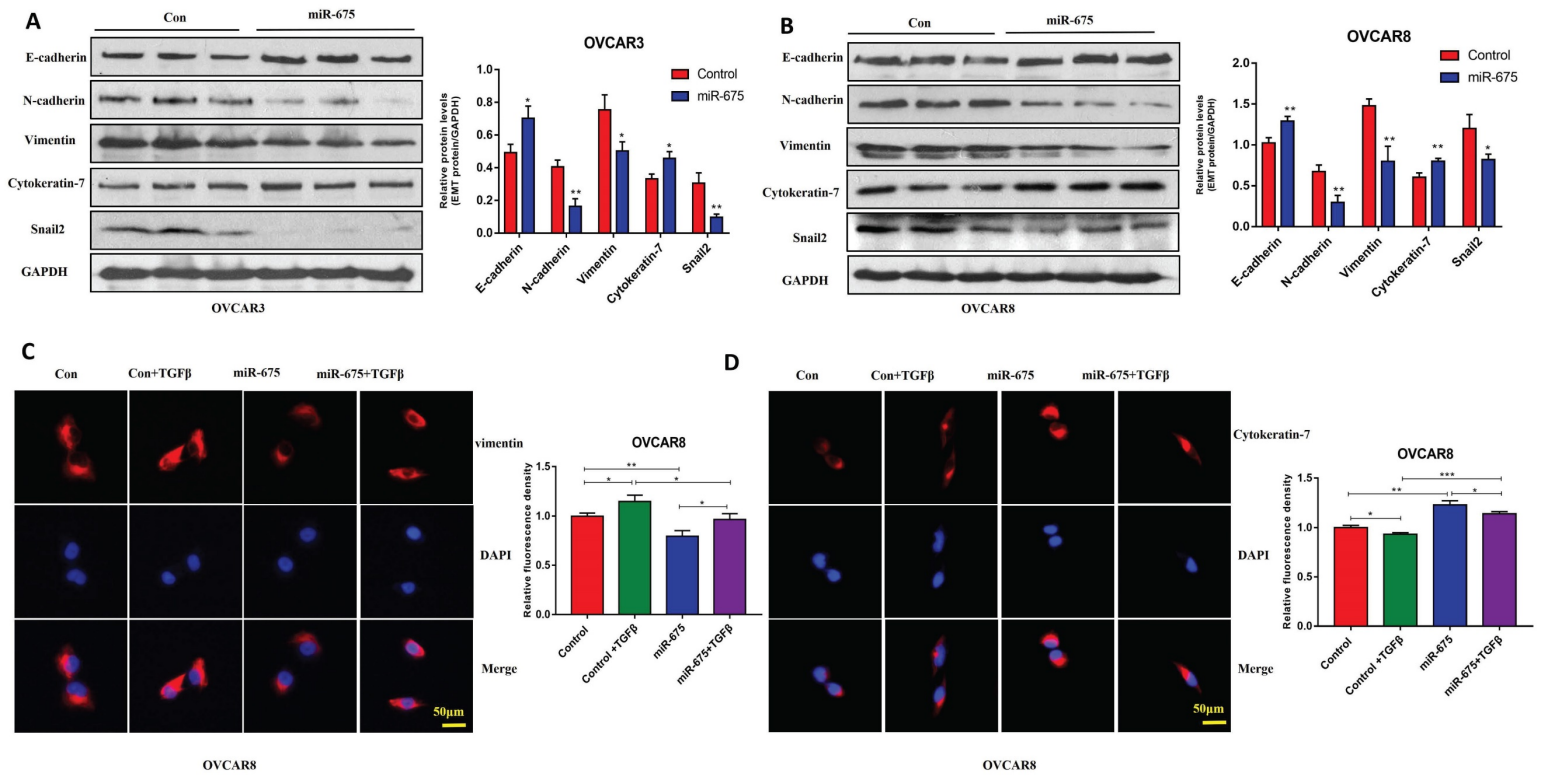
** miR-675 inhibits EMT in OC cells**. **A&B,** Western blot analysis of EMT markers in miR-675-expressing OVCAR3 (A) and OVCAR8 cells (B). **C,** Immunofluorescent staining of vimentin and cytokeratin-7 in miR-675- expressing OVCAR8 cells and controls. * *p* < 0.05; ** *p* < 0.01; *** *p* < 0.001.

To test the effects of miR-675 on EMT in OC cells, we examined EMT markers in miR-675-expressing or control OVCAR3 and OVCAR8 cells. Western blot analysis revealed that miR-675 overexpression led to decreased protein expression of N-cadherin, vimentin, and Snail2, markers associated with the mesenchymal phenotype. Conversely, E-cadherin and cytokeratin-7, markers of the epithelial phenotype, showed increased expression upon miR-675 overexpression (Fig.2A, 2B). To further determine the role of miR-675 in TGFβ induced EMT, we treated both cell lines with 10 ng/ml TGFβ for 24h and then immunostained with vimentin and cytokeratin-7, visually confirming that miR-675 inhibits TGFβ induced EMT markers in OC cells (Fig. 2C, 2D). Overall, these data suggest that miR-675 acts as a negative regulator of EMT in OC cells.

### miR-675 expression inhibits OC cell proliferation, migration and invasion

**Figure 3:**
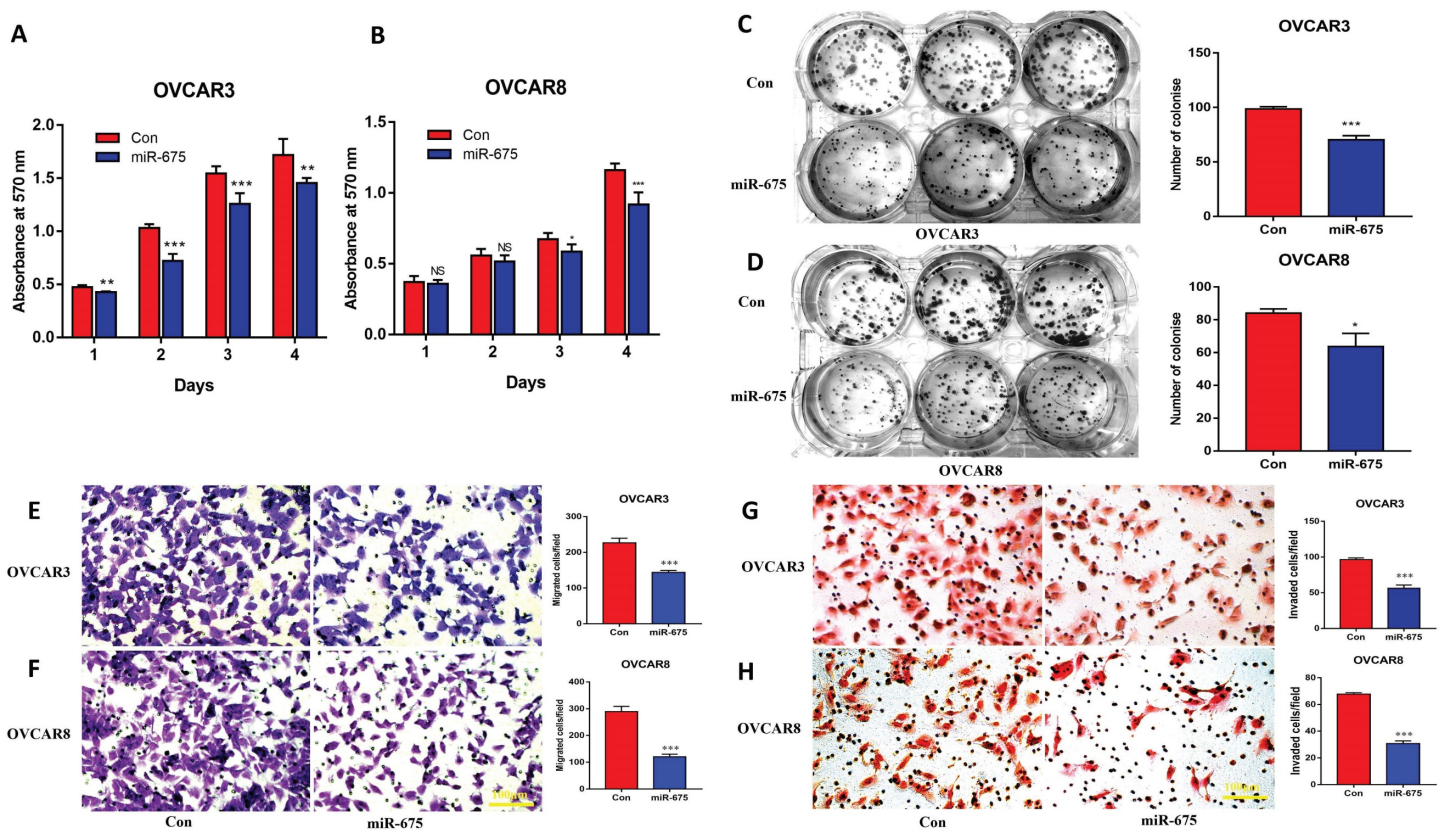
**miR-675 inhibited cell growth, migration, and invasion in OC cells**. **A&B,** Cell proliferation in miR-675-expressing OVCAR3 (A) and OVCAR8 (B) cells compared to controls detected by MTT assay at different time points. **C&D,** Cell colony formation assays were performed to determine cell survival in miR-675-expressing or control OVCAR3 (C) and OVCAR8 (D) cells. **E&F,** Cell migration of miR-675-expressing and control OVCAR3 (E) and OVCAR8 (F) cells. **G&H,** Cell invasion of miR-675-expressing and control OVCAR3 (G) and OVCAR8 (H) cells. * *p* < 0.05; ** *p* < 0.01; *** *p* < 0.001; NS, *p* > 0.05.

To define the functional role of miR-675 in OC, we examined cell proliferation, migration, and invasion in control and miR-675-expressing cells. MTT assays demonstrated that miR-675 overexpression led to reduced cell proliferation in both OVCAR3 and OVCAR8 cells at different time points (Fig. 3A, 3B). These findings were further confirmed by colony formation assays (Fig. 3C, 3D) in both cell lines. Additionally, miR-675 overexpression potently inhibited cell migration (Fig. 3E, 3F) and invasion (Fig. 3G, 3H), suggesting that miR-675 inhibited the metastatic potential of OC cells. These results collectively indicate that miR-675 acts as a tumor suppressor by hindering cell proliferation, migration, and invasion in OC.

### miR-675 expression enhances apoptosis induced by chemotherapy drugs in OC cells

**Figure 4:**
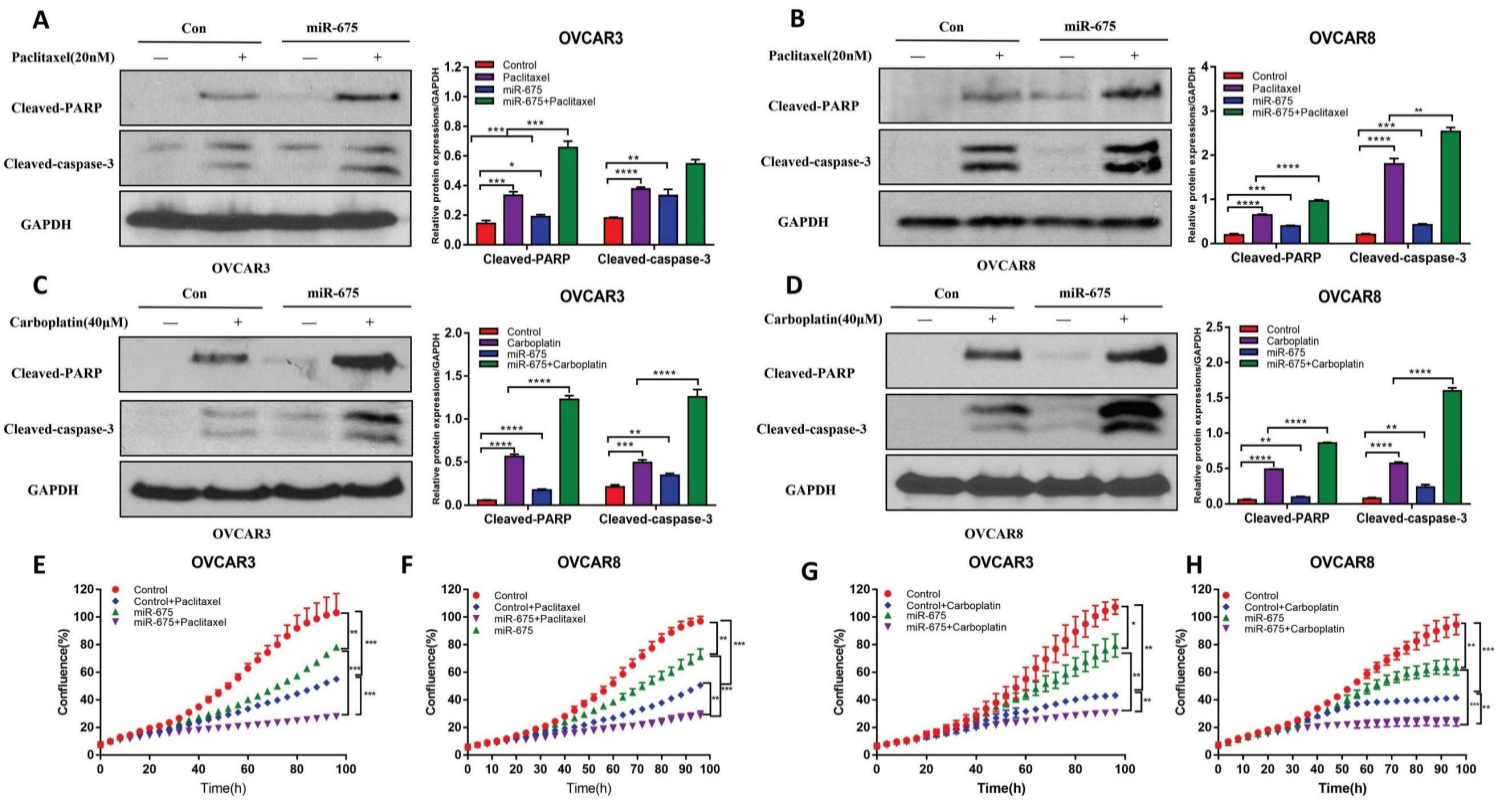
** Overexpression of miR-675 promoted cellular apoptosis**. **A&B,** Apoptosis in miR-675-expressing or control OVCAR3 (A) and OVCAR8 (B) cells treated with paclitaxel or vehicle were examined by detecting cleaved-PARP and Caspase-3 using western blotting. Right panel shows the densitometry of the bands. **C&D,** the expression of cleaved-PARP and caspase-3 was detected using western blotting to assess apoptosis in miR-675-expressing and control OVCAR3 (C) and OVCAR8 (D) cells after treatment with carboplatin or vehicle. Right panel shows the densitometry analysis of the bands. **E&F,** Cell proliferation in miR-675-expressing or control OVCAR3 (E) and OVCAR8 (F) cells with treatment with vehicle or paclitaxel detected by IncuCyte live-cell imaging assays. **G&H,** Cell proliferation in miR-675-expressing or control OVCAR3 (G) and OVCAR8 (H) cells with treatment with vehicle or carboplatin detected by IncuCyte assay. * *p* < 0.05; ** *p* < 0.01; *** *p* < 0.001; **** *p* < 0.0001.

To assess the clinical potential of miR-675 in OC, we tested whether miR-675 expression induces apoptosis or can enhance the apoptosis induced by standard chemotherapeutic drugs. Thus, we treated miR-675-expressing and control OC cells with paclitaxel (20 nM), carboplatin (40 µM) or vehicle for 24h. Cell apoptosis was examined using western blot to detect cleaved-PARP and cleaved-caspase 3. Our data revealed that miR-675 not only induced cell apoptosis, but also displayed a synergistic effect with paclitaxel (Fig. 4A, 4B) or carboplatin (Fig. 4C, 4D). Live cell imaging assays showed the synergistic effects of miR-675 plus paclitaxel (Fig. 4E, 4F) or carboplatin (Fig. 4G, 4H). These data indicate that miR-675 is a potential therapeutic drug which enhanced the effect of conventional chemotherapy agents for OC therapy.

### miR-675 attenuates the TGFβ signaling in OC cells

**Figure 5:**
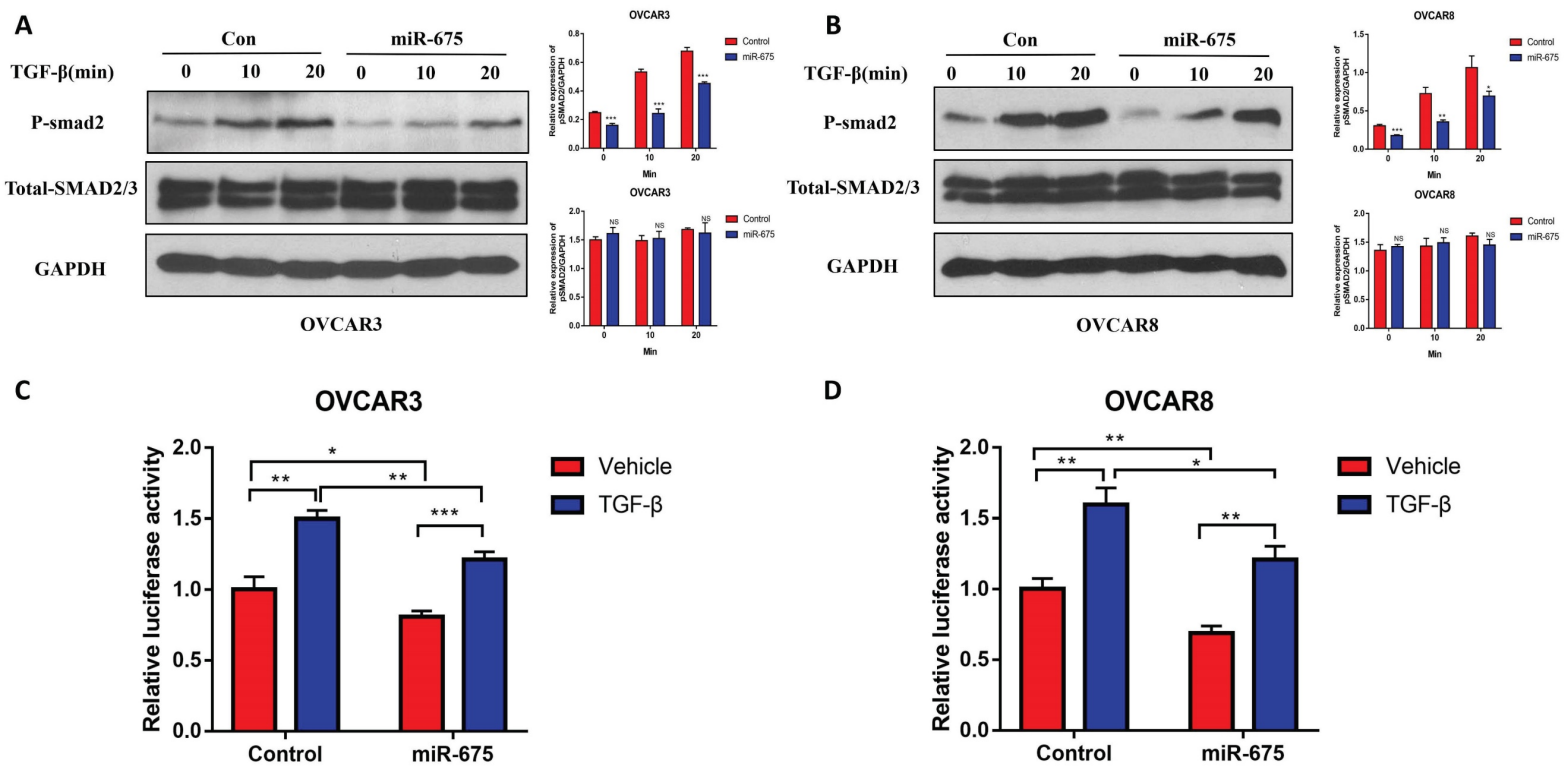
** miR-675 expression attenuates the TGFβ pathway**. **A&B,** Western blot analysis of the p-SMAD2 and total SMAD2/3 expression in both miR-675-expressing or control OVCAR3 (A) and OVCAR8 (B) cells following 10 ng/ml TGFβ treatment at the indicated time points. Right panel shows the densitometry of the bands. **C&D,** Luciferase activity in miR-675-expressing and control OVCAR3 (C) and OVCAR8 (D) cells transduced with pGreenFire1-SMAD2/3/4-GF-EF1-puro lentiviral vector following 10 ng/ml TGFβ treatment for 24 h. * *p* < 0.05; ** *p* < 0.01; *** *p* < 0.001; NS, *p* > 0.05.

We previously demonstrated that TGFβ induces EMT in OC cells 35. Since miR-675 has been reported to target TGFβ or its receptor 45, 46, we investigated whether miR-675 inhibits EMT by attenuating the TGFβ signaling pathway. We treated both miR-675-expressing and control OVCAR3 and OVCAR8 cells with 10 ng/ml TGFβ for various time points. Western blot analysis revealed that miR-675 overexpression significantly reduced the levels of phosphor-SMAD2 (pSMAD2) in both cell lines, while total SMAD2/3 protein levels remained largely unchanged (Fig. 5A, 5B). We further examined the effects of miR-675 on downstream effectors of the TGFβ pathway. We transduced both miR-675-expressing and control cells with a reporter vector containing SMAD2/3/4 transcriptional response elements (TRE-CMV-Luc) and then treated the cells with 10 ng/ml TGFβ for 24 hours. Luciferase activity (measure of SMAD2/3 transcriptional activity) was significantly inhibited by miR-675 expression in both OVCAR3 (Fig. 5C) and OVCAR8 (Fig. 5D) cells. These data collectively suggest that miR-675 acts as a negative regulator of the TGFβ signaling pathway in OC cells.

### miR-675 inhibits primary tumor growth and metastasis in an orthotopic OC mouse model

**Figure 6:**
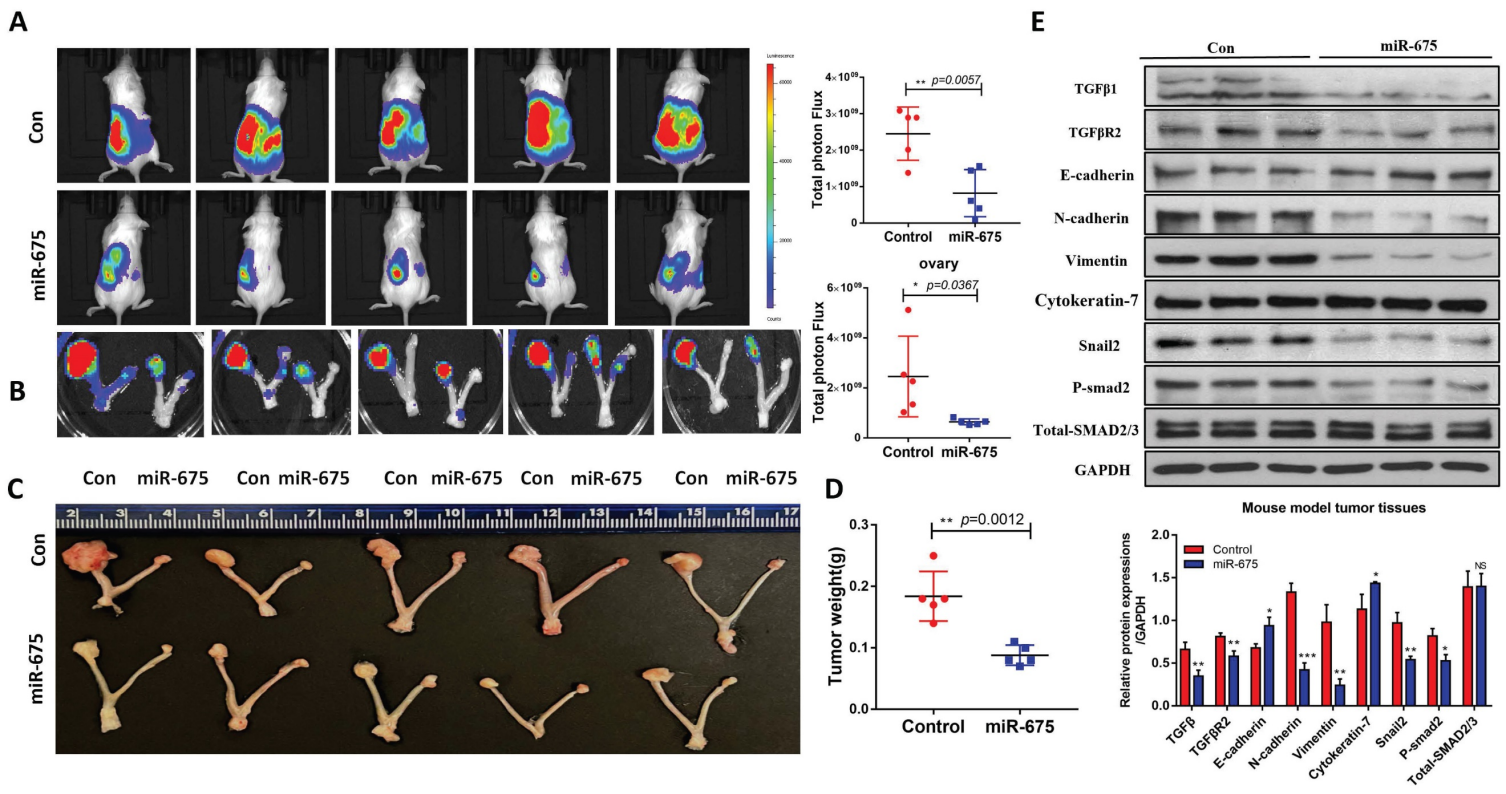
** miR-675 overexpression inhibits primary ovarian tumor growth in an orthotopic OC mouse model. A,** Bioimaging of mice at 4 weeks following intrabursal injection of miR-675-expressing and control OVCAR8 cells. Right panel shows the quantitative analysis. **B,** Primary tumors in ovaries of mouse were imaged by live animal imaging when collecting tissues. Right panel shows the quantitative analysis. **C,** Tumors in ovaries of mice were dissected, imaged. **D,** Tumor wet weight in ovaries. **E,** Western blot analysis shows the expression of TGFβ1, TGFBR2, p-smad2, Total-SMAD2/3, and EMT markers in primary ovarian tumors. Lower panel shows the densitometry analysis of the bands. * *p* < 0.05; ** *p* < 0.01; *** *p* < 0.001; NS, *p* > 0.05.

**Figure 7:**
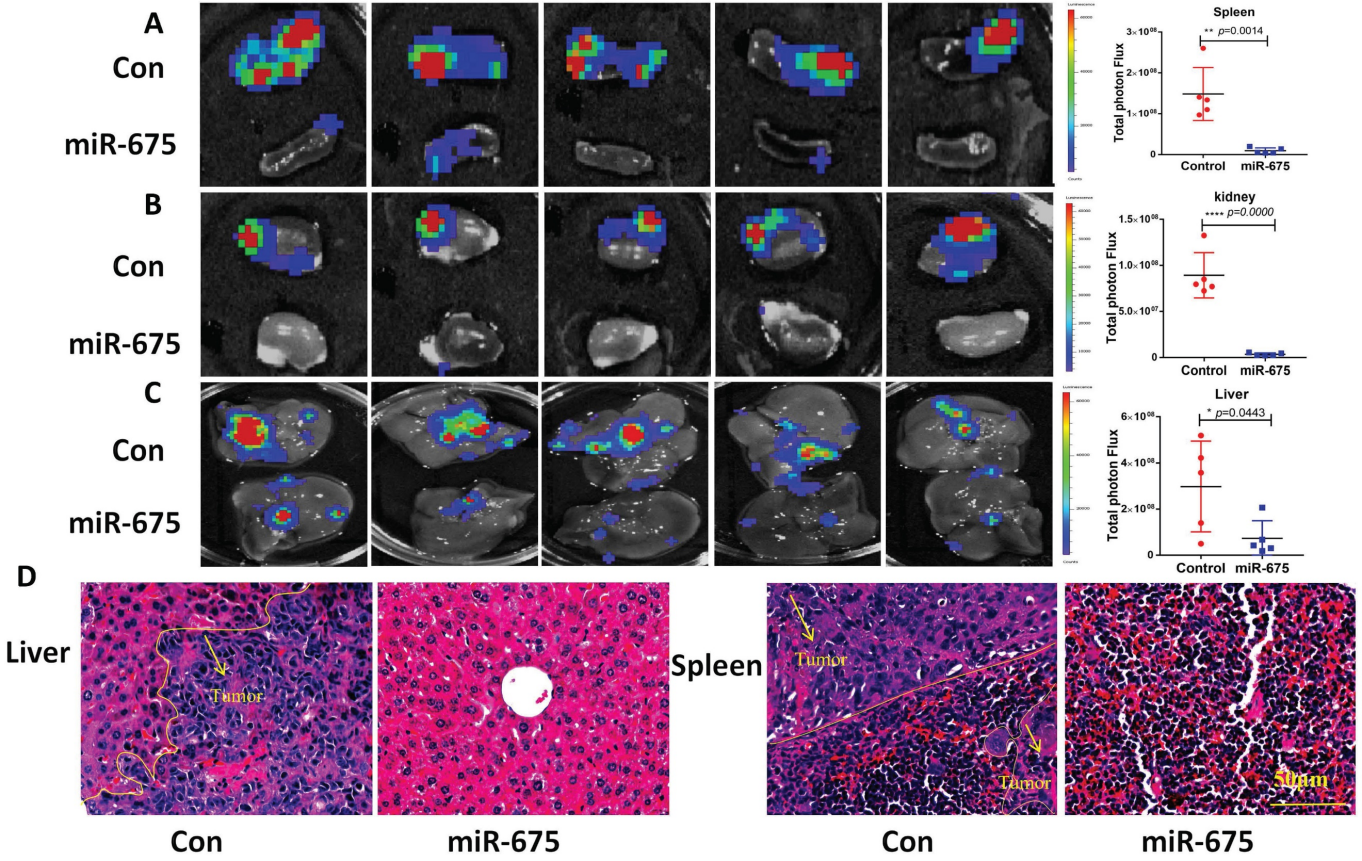
** miR-675 overexpression inhibits primary ovarian tumor metastasis in an orthotopic OC mouse model. A&B&C,** Metastatic tumors in spleen (A), kidney (B), and liver (C) of xenografted mice intrabursally injected with miR-675-expressing and control OVCAR8 cells were identified by live animal imaging. Right panels show the quantitative analysis. D, H&E staining of liver and spleen from mice injected with control (Con) or miR-675-expressiong OVCAR8 cells. Arrows indicate the area of tumor. * *p* < 0.05; ** *p* < 0.01; **** *p* < 0.0001.

Our *in vitro* findings demonstrated that miR-675 acts as a tumor suppressor by inhibiting EMT and attenuating the TGFβ signaling pathway in OC cells. We then examined whether miR-675 suppresses ovarian cancer growth and metastasis *in vivo*. Using an orthotopic OC mouse model as described before 48, miR-675-expressing and control OVCAR8 cells stably expressing luciferase were injected intrabursally into female NSG mice. Tumor growth in the ovaries and metastatic spread to distant organs were monitored using bioluminescence imaging after D-luciferin injection every week, after 4 weeks, the mice were sacrificed, and tissue were collected. The bioluminescent signal intensity at 4 weeks indicated a significant reduction in primary ovarian tumor burden in mice injected with miR-675-expressing OVCAR8 cells compared to the control group (Fig. 6A, 6B). This finding was further corroborated by significantly reduced tumor size and weight (Fig. 6C, 6D). Western blot analysis of primary ovarian tumors revealed that miR-675 expression led to the upregulation of epithelial markers cytokeratin-7 and E-cadherin, while mesenchymal markers N-cadherin, Vimentin, and Snail2 were downregulated. Additionally, TGFβ1, TGFBR2, and p-SMAD2 levels were decreased (Fig. 6E). Furthermore, we examined tumor metastasis in multiple peritoneal organs. While tumors were found in the spleens, kidneys, and livers of control mice, metastasis was significantly reduced in mice injected with miR-675-expressing OVCAR8 cells, with tumors detected only in a few mice (Fig. 7A, 7B, 7C). H&E staining of liver and spleen further confirmed our findings (Fig. 7D).

**Figure 8:**
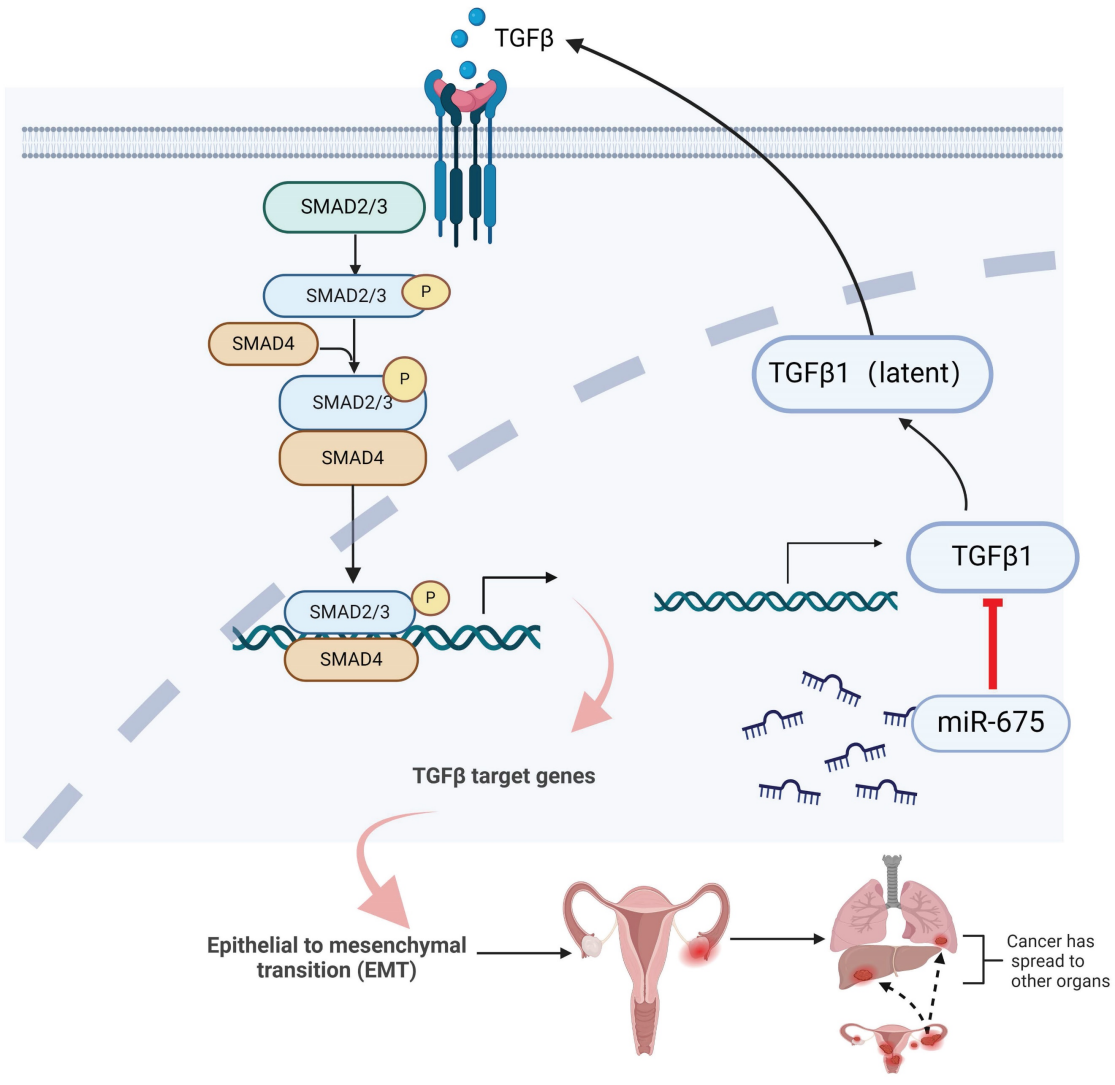
** Summary of inhibition of miR-675 on TGFβ signaling and OC progression.** miR-675 disrupts key processes involved in OC progression and metastasis by targeting the TGFβ signaling pathway and EMT. This figure was created with BioRender.com.

Taken together, these findings demonstrate that miR-675 expression suppresses primary ovarian tumor growth and metastasis in part by targeting the TGFβ1, inhibiting EMT through attenuating the TGFβ pathway (Fig. 8), suggesting the potential of miR-675 as a therapeutic strategy for OC patients.

## Discussion

This study unveils a promising role for miR-675 as a potent suppressor of OC progression. Our findings demonstrate, for the first time, that miR-675 overexpression significantly inhibits primary tumor growth and metastasis in both *in vitro* and *in vivo* OC models. This tumor-suppressive effect is mediated by miR-675's ability to attenuate the TGFβ signaling pathway and suppress EMT, a key driver of metastasis. Our data also suggest that miR-675 showed synergistic effect with standard chemotherapeutic drugs, potentially improving treatment efficacy.

We observed that miR-675 overexpression resulted in elevated expression of both miR-675-5p and miR-675-3p. Interestingly, endogenous expression of both miR-675-5p and 3p was lower in the more aggressive OVCAR8 cells compared to OVCAR3 cells. This finding suggests a potential correlation between low miR-675 expression and the aggressive phenotype of OVCAR8 cells. Although miR-675 is processed from LncRNA-H19, miR-675 functions as a tumor suppressor in OC is confirmed in this study. LncRNA-H19 was previously identified as an oncogene and promotes EMT by sponging miR-140-5p in OC 49. Our studies indicated that miR-675 acts as tumor suppressor in OC, which is independent of LncRNA-H19's function as an oncogene in OC. Although we did not investigate miR-675-5p or miR-675-3p individually, miR-675-5p is more robustly expressed than miR-675-3p in OC cells following transduction. Therefore, the tumor suppressor role of miR-675 may represent the effects of both miR-675-5p and 3p in OC. Further investigations are needed to delineate the specific contributions of miR-675-5p and miR-675-3p to the observed tumor-suppressive effects in OC.

We previously reported that TGFβ induced EMT in OC cells 50. Our data provide strong evidence that miR-675 exerts its anti-tumorigenic effects by targeting the TGFβ signaling pathway and suppressing EMT. This is supported by the downregulation of pSMAD2 upon miR-675 overexpression. Due to attenuation of the TGFβ pathway in miR-675 expressing cells, we hypothesized that miR-675 targets components of the TGFβ pathway, based on previous identification of TGFβ1 and TGFBR1 as targets of miR-675 45. We examined both TGFβ1 and TGFBR2 in both miR-675 expressing and control OC cells and found that miR-675 downregulated both TGFβ1 and TGFBR2, which may be a mechanism underlying the miR-675 suppressor role. Our orthotopic models also validated the finding that miR-675 inhibited OC growth and metastasis by suppressing EMT and the TGFβ pathway. There are multiple potential targets of miR-675 and downstream pathways in OC cells. Thus, further studies are required to fully demonstrate targets of miR-675 in its function as a tumor suppressor.

The synergistic effect of miR-675 with paclitaxel and carboplatin on apoptosis induction indicates its potential for combination therapy strategies by inducing cell apoptosis. miR-675 may improve clinical therapy for OC by sensitizing cell responses to chemotherapy agents. Our data also suggest that miR-675 may overcome chemoresistance by suppressing EMT. Further investigations are warranted to explore the feasibility and efficacy of different delivery methods of miR-675 for clinical applications. Additionally, elucidating the precise downstream targets of miR-675 and its interactions with other signaling pathways could provide valuable insights for targeted therapeutic development. This study primarily focused on the tumor-suppressive effects of miR-675 in established OC cell lines and an *in vivo* mouse model. Future studies should explore the role of miR-675 in different stages and histological subtypes of OC and chemoresistance including miR-675-5p and 3p.

In conclusion, our study establishes miR-675 as a promising candidate for novel therapeutic strategies in OC. By targeting the TGFβ signaling pathway and EMT, miR-675 disrupts key processes involved in tumor progression and metastasis. The encouraging preclinical data and potential for combination therapy with established drugs indicate miR-675 as a therapeutic target for OC patients.
